# Metastatic Sternal Osteosarcoma: A Rare Tumor

**DOI:** 10.7759/cureus.2206

**Published:** 2018-02-19

**Authors:** Muhammad Masab, Ena Arora, Sorab Gupta, Hafsa Farooq, Vishal Jindal, Shorabh Sharma

**Affiliations:** 1 Internal Medicine, Albert Einstein medical center; 2 Internal Medicine, Government Medical College and Hospital Chandigarh; 3 Hematology/oncology, Albert Einstein Medical Center; 4 Internal Medicine, Waterbury Hospital; 5 Internal Medicine, St. Vincent Hospital Worcester; 6 Internal Medicine, SBH Health System

**Keywords:** osteosarcoma, chest wall tumor, sternal tumor, pulmonary metastasis

## Abstract

Osteosarcoma is the most common primary malignant tumor of the long bones. However, primary osteosarcoma of the chest wall, particularly the sternum, is an extremely rare occurrence. We report a 36-year-old male presenting with a hard, immobile, palpable, anterior chest wall mass. A computed tomographic (CT) scan showed a large destructive anterior mediastinal mass involving the manubrium and sternum with multiple bilateral calcified lung masses, pleural effusions and partially calcified aortopulmonary, right hilar and subcarinal lymphadenopathy. Incisional biopsy of the mass revealed grade 2 chondroblastic osteosarcoma. The patient underwent one cycle of chemotherapy with ifosfamide and palliative radiation. Unfortunately, the patient was unable to tolerate ifosfamide and developed severe nausea and vomiting requiring the discontinuation of chemotherapy. Given his metastatic disease and inability to tolerate standard chemotherapy, he was referred to a comprehensive cancer center for advanced clinical trials.

## Introduction

Primary osteosarcoma of the sternum is an extremely rare tumor, with a reported median age of 42 years at the time of diagnosis [1]. It is considered an aggressive malignant tumor with poor prognosis [2]. We present a unique case of primary osteosarcoma of the sternum with metastasis to the lungs. Osteosarcoma should always be considered in the differential diagnosis of an anterior chest wall mass. Given the aggressive nature of this tumor and the poor prognosis associated with metastatic disease, early diagnosis and management are of paramount importance.

## Case presentation

**Figure 1:**
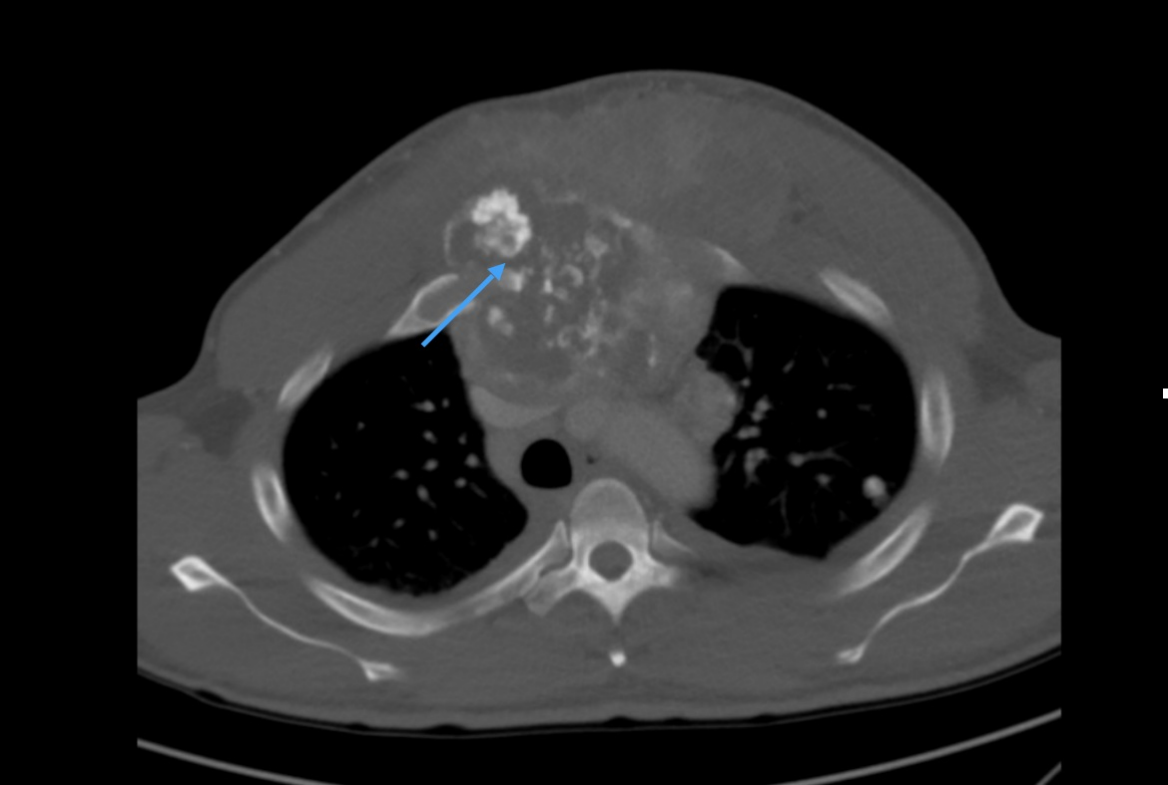
Computed tomography (CT) of the chest with contrast revealing a 11.5 x 15.9 x 11.9 cm anterior calcified mediastinal mass (blue arrow) involving the manubrium and sternum, extending anteriorly into the pectoral muscles

A 36-year-old African American male with no significant past medical history presented to our cancer center with a six-month history of an anterior chest wall mass. His other symptoms included chest pain, cough, and an unintentional weight loss of 5 lbs over a period of one month. The physical exam was significant for a hard, palpable mass on the anterior chest overlying the sternum, fixed to the chest wall and extending 20 cm horizontally and 14 cm vertically. The laboratory analysis was significant for mild anemia with Hb 12.7 g/dl (13.5-17.5 g/dl), elevated lactate dehydrogenase (LDH) with a value of 908 U/L (140-260 U/L), and elevated alkaline phosphatase (ALP) with a value of 418 IU/L (44-147 IU/L). A computed tomography (CT) scan of the chest and abdomen with contrast revealed a large (11.5 x 15.9 x 11.9 cm) destructive anterior mediastinal mass involving the manubrium and sternum, extending anteriorly into the pectoral muscles (Figure 1).

**Figure 2:**
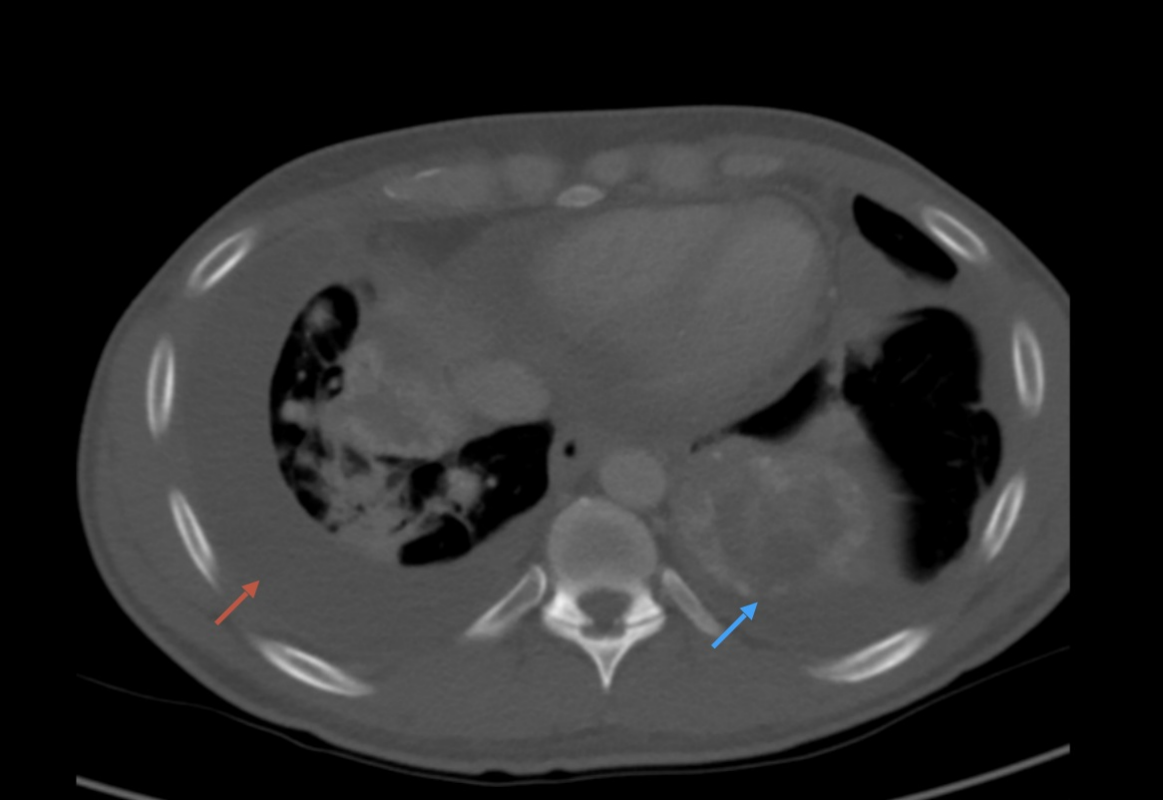
Computed tomography (CT) of the chest with contrast illustrating the bilateral pulmonary metastatic lesions with a large left lower lobe lung mass (blue arrow), bilateral pleural (red arrow) and pericardial effusions

It also showed multiple bilateral calcified lung masses, the largest one located in the left lower lobe measuring 6.3 x 5.1 cm (Figure 2).

There were bilateral pleural effusions, pericardial effusion and partially calcified aortopulmonary, right hilar and subcarinal lymphadenopathy. He underwent diagnostic thoracentesis which was negative for malignant cells, and incisional biopsy of the mediastinal mass that came back positive for grade 2 chondroblastic osteosarcoma. He also underwent biopsy of the rapidly growing lung lesion which was consistent for chondroblastic osteosarcoma. He was started on first-line chemotherapy with single-agent ifosfamide and underwent a brief course of palliative radiation therapy. Unfortunately, the patient was unable to tolerate ifosfamide and developed severe nausea and vomiting requiring the discontinuation of chemotherapy. He was planned to start on cisplatin and adriamycin but refused further treatment as he was worried about serious side effects. Given his metastatic disease and inability to tolerate standard chemotherapy, he was referred to a comprehensive cancer center for advanced clinical trials.

## Discussion

Chest wall tumors are classified into four main groups: primary, metastatic, extension from adjacent structures or non-neoplastic lesions. Primary chest wall osteosarcoma is a rare entity and constitutes 10% of all primary chest wall tumors. It accounts for almost 3% of all sarcomas. It is considered an aggressive malignant tumor with poor prognosis. The occurrence of osteosarcoma in the sternum is extremely rare, with a reported median age of 42 years at the time of diagnosis [1]. 

Patients with osteosarcoma of the sternum typically present with a rapidly enlarging painful mass, with or without a history of radiation exposure. In many cases, they may also be completely asymptomatic. These tumors do have a metastatic potential and it is reported that 34% of patients have metastasis at the time of presentation [1]. Osteosarcomas present both as locally advanced disease as well as with distant metastasis. The initial workup includes imaging of the primary site (plain radiographs, CT and/or magnetic resonance imaging (MRI)), and additional imaging for staging purpose including positron emission tomography (PET), CT, and bone scan.

Plain radiographs of osteosarcoma show irregular reactive bone and cortical destruction. A bone scan may show additional synchronous lesions. An MRI provides excellent soft-tissue contrast and may be essential for operative planning. It is the best imaging modality to define the extent of the lesion within the bone, as well as the extra osseous spread of tumor including soft tissue extension and “skip” metastases. In addition, ALP and LDH levels are frequently elevated in patients with osteosarcoma, especially with metastatic disease [3].

Management depends on the extent of the disease. Preoperative chemotherapy followed by wide excision of the primary tumor is recommended for localized disease. Chemotherapy and metastasectomy (pulmonary, visceral or skeletal) are included as options for the management of metastatic disease. An unresectable metastatic disease requires management with chemotherapy and/or radiation therapy.

Although chemotherapy is associated with improved outcomes in patients with localized osteosarcoma, the results are significantly poorer in patients with metastatic disease at presentation, resulting in a very poor prognosis [4]. Bacci et al. studied 44 patients with metastatic osteosarcoma (with pulmonary metastasis) who received neoadjuvant chemotherapy followed by surgery and adjuvant chemotherapy. The five-year overall survival and disease-free survival rates for all 44 patients were 14% and 17% respectively. These were much worse than those achieved with the same chemotherapy protocol in 144 patients with localized disease at presentation (79% overall survival and 73% disease-free survival) [5].

In a study of 57 patients with metastatic disease at presentation treated with cisplatin, doxorubicin, and high dose of methotrexate and ifosfamide, the two-year event-free survival and overall survival rates were 21% and 55%, respectively, compared to 75% and 94% in patients with non-metastatic disease at presentation, treated with the same chemotherapy protocol [6]. Among patients with primary metastases treated in cooperative osteosarcoma trials, long-term survival rates were higher for patients whose metastases were excised following chemotherapy and surgery of the primary tumor compared to those patients with unresectable metastases (48% and 5% respectively).

## Conclusions

This case is presented to highlight the diagnosis and management of chest wall osteosarcoma. Primary osteosarcoma of the sternum is a rare tumor. The physicians should always consider the possibility of a malignancy in patients presenting with an anterior chest wall mass. Given the aggressive nature of the tumor and poor prognosis, early recognition and prompt treatment are critical.
